# Comparative Genomics of *Spatholobus suberectus* and Insight Into Flavonoid Biosynthesis

**DOI:** 10.3389/fpls.2020.528108

**Published:** 2020-09-04

**Authors:** Shuangshuang Qin, Kunhua Wei, Zhanhu Cui, Ying Liang, Mingjie Li, Li Gu, Chuyun Yang, Xiaolei Zhou, Linxuan Li, Wei Xu, Can Liu, Jianhua Miao, Zhongyi Zhang

**Affiliations:** ^1^ College of Agriculture, Fujian Agriculture and Forestry University, Fuzhou, China; ^2^ Guangxi Key Laboratory of Medicinal Resources Protection and Genetic Improvement, Guangxi Botanical Garden of Medicinal Plants, Nanning, China; ^3^ Center for Research and Cooperation, Novogene Bioinformatics Institute, Beijing, China; ^4^ Key Laboratory of Genetics, Breeding and Comprehensive Utilization of Crops, Ministry of Education, Fujian Agriculture and Forestry University, Fuzhou, China

**Keywords:** *Spatholobus suberectus* Dunn, comparative genome analysis, flavonoid biosynthesis, transcription factors, isoflavone synthase

## Abstract

*Spatholobus suberectus* Dunn (*S. suberectus*), has been widely used in traditional medicines plant source of the Leguminosae family. Its vine stem of which plays an important role in the prevention and treatment of various diseases because it contains various flavonoids. Comparative genome analysis suggested well-conserved genomic components and genetic collinearity between the genome of *S. suberectus* and other genera of Leguminosae such as *Glycine max*. We discovered two whole genome duplications (WGD) events in *S. suberectus* and *G. max* lineage underwent a WGD after speciation from *S. suberectus.* The determination of expansion and contractions of orthologous gene families revealed 1,001 expanded gene families and 3,649 contracted gene families in the *S. suberectus* lineage. Comparing to the model plants, many novel flavonoid biosynthesis-related genes were predicted in the genome of *S. suberectus*, and the expression patterns of these genes in the roots are similar to those in the stems [such as the isoflavone synthase (*IFS*) genes]. The expansion of *IFS* from a single copy in the Leguminosae ancestor to four copies in *S. suberectus*, will accelerate the biosynthesis of flavonoids. *MYB* genes are widely involved in plant flavonoid biosynthesis and the most abundant member of the TF family in *S. suberectus*. Activated retrotransponson positive regulates the accumulation of flavonoid in *S. suberectus* by introducing the cis-elements of tissue-specific expressed *MYBs*. Our study not only provides significant insight into the evolution of specific flavonoid biosynthetic pathways in *S. suberectus*, but also would facilitate the development of tools for enhancing bioactive productivity by metabolic engineering in microbes or by molecular breeding for alleviating resource shortage of *S. suberectus*.

## Introduction

**Figure 1:**
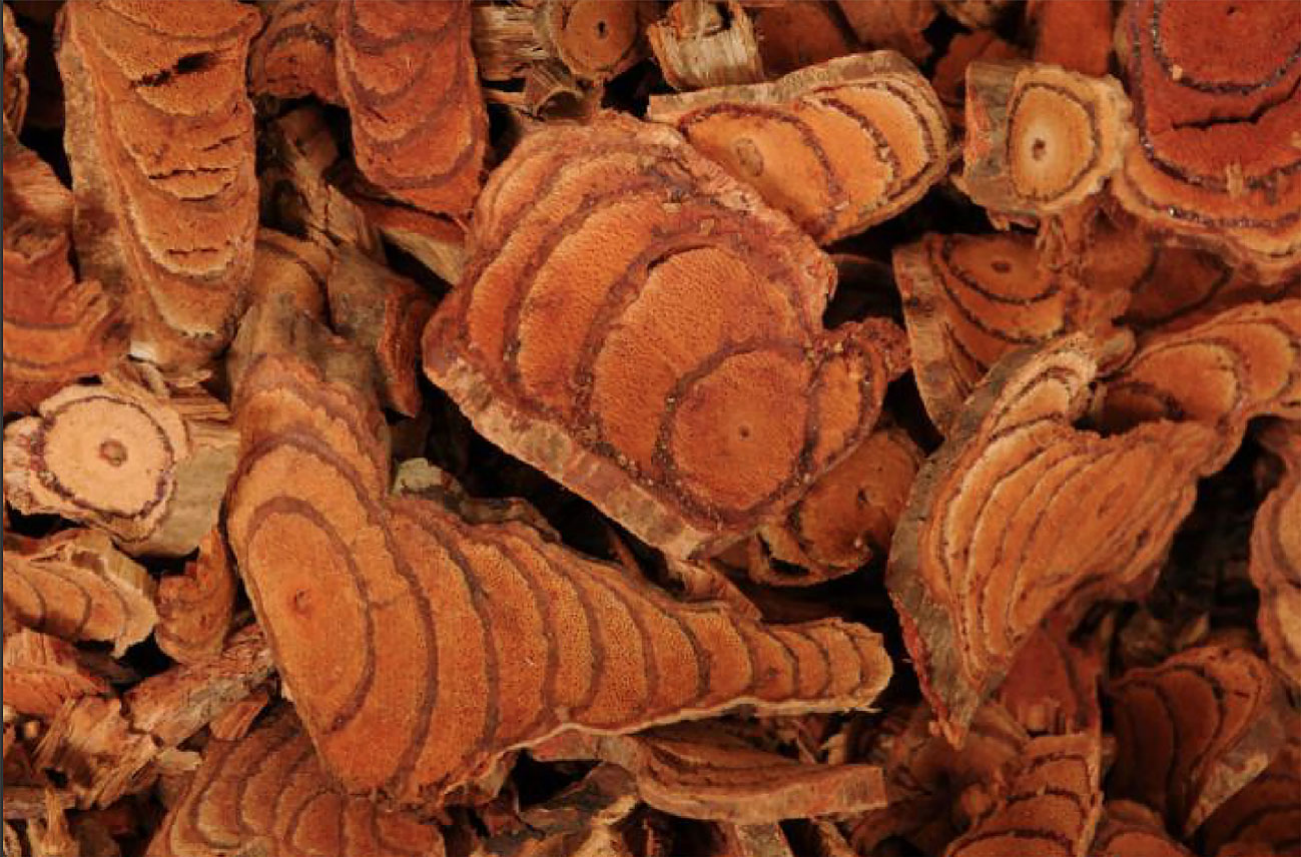
The dry stem of *Spatholobus suberectus*.

*Spatholobus suberectus* Dunn (*S. suberectus*) is an Leguminosae popularly used in Chinese Traditional Medicine. Pharmacological and clinical studies have demonstrated that the dried stems of *S. suberectus* (**Figure 1**) exhibit various functions and flavonoids are the main bioactive components (Wang et al., 2011; Zhou et al., 2017). Four flavonoid compounds have been found to have important pharmacological activities, among which formononetin, genistein, and isoliquiritigenin are effective in cancer prevention or therapy (Wang et al., 2011; Peng et al., 2016) and catechin can promote the proliferation of hematopoietic progenitor cells (Wang et al., 2008). *S. suberectus* is therefore widely used in patented Chinese medicines, and the market demand for the wild resource is increasing rapidly. In addition, the crud drug of *S. suberectus* must grow for more than 7 years before it can be used in medicine. Owing to its long growth cycle and increased use in medicines, the wild resources of *S. suberectus* in China are on the verge of extinction.

Comparative genome analysis is an effective means of investigating the evolution and identifying functional elements of *S. suberectus* genome. Based on the reported genome sequence of *S. suberectus* with 798 Mb in size (Qin et al., 2019), we compared it with the genomes of other reported leguminous plants, such as *Lotus japonicus* (Sato et al., 2008), *Glycine max* (Schmutz et al., 2010), *Medicago truncatula* (Young et al., 2011), *Glycyrrhiza uralensis* (Mochida et al., 2017), and *Cicer arietinum* (Gupta et al., 2017), to determine how genomes came to be and for the study of evolution. Whole-genome duplication (WGD) events, which create additional copies of the entire genomes in organisms, have a considerable influence on plant evolution and speciation.

Although many genes involved in the flavonoids biosynthesis have been identified (Bowerman et al., 2012; Saito et al., 2013), the overall genetic information of the flavonoids involved in biosynthetic pathways of *S. suberectus* remains lacking. Transcription factors, which have great values in flavonoid biosynthesis, have not been studied for *S. suberectus.* The expansion of some genes are likely related to its abundance of compounds and their expression are just correlate with content of the main bioactive components in this study. These results will be value for biosynthesis studies seeking to the rapid accumulation of bioactive components by metabolic engineering in microbes or by molecular breeding for alleviating resource shortage of *S. suberectus*.

## Methods

### Plant Materials

*S. suberectus* plants were grown in Guangxi Botanical Garden of Medicinal Plants (22°51’28” N, 108°22’2” E). Plant tissues, including roots, stems, leaves, flowers, and fruits from 8-years-old plants were collected. Each plant tissue had three biological repetitions. Each sample was randomly collected from five plants and divided into three groups for different purposes: quick-frozen samples, used for RNA isolation; dried samples, used for total flavonoid content determination; and freeze-dried samples, used for bioactive compounds content measurement.

### Comparative Genome Analysis

Comparative analysis was performed to identify orthologous gene families among the eight plant species as described in the main text including *S. suberectus*. For all-against-all proteins blast, we first filter the proteins with length less than 50 amino acids and retained the longest protein with alternative splicing variations, then using the BLASTP (E value < le−7) to blast the filtered proteins and clustered into orthologous groups using OrthoMCL with the inflation parameter at 1.5 (Li et al., 2003). One protein per species in a cluster was clustered into the single-copy orthologues, which were used for MUSCLE alignment and generated the phylogenetic tree *via* the maximum likelihood method (Edgar, 2004). The divergence time of each species was generated *via* the PAML MCMCtree (http://abacus.gene.ucl.ac.uk/software/paml.html) with the two corrected divergence time point, *A. thaliana* *vs*. *G. max*: 97~109 Mya; *A. thaliana* *vs*. *Salvia miltiorrhiza*: 110~124 Mya, from the TimeTree (http://http://www.timetree.org). The gene families’ expansion or contraction analysis were conducted by the CAFÉ software. The whole genome duplication (WGD) analysis was performed by the MCscanX software with default parameters.

### Transcriptome Library Preparation and Gene Expression Analysis

Three technical replicates for each sample and prepared for RNA isolation and transcriptome library construction. Total RNAs were extracted using TRIzol^®^ Reagent (Thermo Fisher Scientific, USA) according to the manufacturer’s instructions, while the RNA-seq libraries were constructed using the NEBNext Ultra Directional RNA Library Prep Kit (NEB, USA). The raw data were then filtered according the standard quality control (QC) method. The retained pared end reads were then mapped to the *S. suberectus* reference genome using HISAT2 (http://ccb.jhu.edu/software/hisat2/index.shtml). The total numbers of aligned reads were normalized by gene length and sequencing depth for an accurate estimation of expression level and then used the Reads Per Kilobase per Million mapped reads (RPKM) to represent the expression level of each gene for further calculation of the differential expression gene (DEG) using Deseq2 software (https://bioconductor.org/packages/release/bioc/html/DESeq2.html).

We used these normalized read counts (RPKM) as the expression level for each gene.

Based on log10 (RPKM+1) values, we used the R software (version 3.3.1) to plot the heatmap graph with package heatmap.

### Identification of the Candidates in Flavonoid Biosynthesis Pathways

In order to detect the candidate genes in the flavonoid biosynthesis pathways in the Leguminosae species, including *S. suberectus*, *G. max*, *L. japonicus*, *G. uralensis*, *C. arietinum*. The similarity calculated by BLASTP searching (e-value <= 1e−5) with known members from the model species G. max and A. thaliana and other reported plants, we have searched 15 genes involved in flavonoid synthesis in the above genome, including phenylalanine ammonia-lyase (PAL), cinnamate-4-hydroxylase (C4H, a CYP450 gene) and 4-coumarate CoA ligase (4CL), the first three enzymes in phenylpropanoid pathway (Saito et al., 2013), and others enzymes in this pathway including chalcone synthase (CHS), chalcone isomerase (CHI) (Saslowsky and Winkel-Shirley, 2001), isoflavone synthase (IFS, a CYP450 gene), 2-hydroxyisoflavanone dehydratase (HID) (Jung et al., 2000; Shimamura et al., 2007), flavanone-3-hydroxylase (F3H, a 2-OGD gene) (Pelletier and Shirley, 1996), flavanone-3’-hydroxylase (F3’H, a CYP450 gene) (Schoenbohm et al., 2000), dihydroflavonol 4-reductase (DFR) (Shirley et al., 1992), anthocyanidin synthase (ANS, alias LDOX, a 2-OGD gene) (Bowerman et al., 2012), anthocyanidin reductase (ANR) (Devic et al., 1999), flavonol synthase (FLS, a 2-OGD gene) (Pelletier et al., 1997), leucoanthocyanidin reductase (LAR) (Tanner et al., 2003), O-methyltransferase (OMT) (Hashim et al., 1990; Dhaubhadel et al., 2003; Li et al., 2016), and chalcone reductase (CHR, alias PKR, also named PKR) (Shimada et al., 2005). Because the homology of different genes is different, we choose different Identity thresholds. PAL, 4CL, CHS, CHI, HID, LAR, OMT, and ANR identity>=45; ANS, FLS, F3H (belonging to 2-OGD), C4H, F3’H (belonging to CYP450), DFR, CHR (belonging to reductase) identity>=65.

Because of the *IFS* gene were treated as repeat sequences be masked, the pipeline determined IFS protein sequences from the originally predicted coding sequence by gene wise with protein sequences of *G. max* as reference, combine with the AUGUSTUS software to predict the structure. For phylogenetic analyses, the total IFS proteins from five Leguminosae plants (including *S. suberectus*, *G. max*, *L. japonicus*, *G. uralensis*, and *C. arietinum*) were subject to do multiple alignments using MUSCLE. Neighbor-joining tree was built using TreeBeST with the Jones–Taylor–Thornton (JTT) model and 100 bootstrap replicates using results of multiple alignments. We analyzed structure of the *IFS* genes among five Leguminosae plants and investigated the position relationship between the repetitive element and the *IFS* genes.

### Total Flavonoid Content

Determination of total flavonoid content (TFC): The TFC was carried out according to Chen et al. (2016). Dry samples (0.3 g) and sonicated in 25 ml of 50% ethanol at a ratio of 1:20 (w/v) for 1 h using a SB-800 DTD sonicator (Ningbo Xinzhi Biotechnology Co., Ltd, Ningbo, China; power: 100 W; frequency: 40 kHz). In this method, rutin was used as standard and flavonoid contents were measured as rutin equivalent. For this purpose, the calibration curve of rutin was drawn. One milliliter of standard or extract solution (0.5, 1.0, 1.5, 2.0, 2.5, 3.0 ml) was taken into 25 ml volumetric flask, and 1 ml of 5% NaNO_2_ added to the flask. After 5 min, 1 ml 10% AlNO_3_ was added to the mixture. At the 5th min add 10 ml of 4% NaOH was added and volume made up to 25 ml with 50% ethanol. The absorbance was noted at 505 nm using UV-Visible spectrophotometer.

### Ultraperformance Liquid Chromatography-Electrospray Ionization-Mass Spectrometry/Mass Spectrometry Analysis

Each standard compound was accurately weighed, and then dissolved in methanol–water (80:20, v/v) solvent to a final diluted stock solutions of 100 μg/ml. Working standard solutions containing five reference standards were prepared by diluting the stock solutions with methanol–water (80:20, v/v) solvent to produce the standard curves. The solutions were stored at 4°C for further analysis.

All the freeze-dried samples were cut into smaller pieces, further grounded into powder. Each sample powder (0.05 g) was weighed accurately soaked in 1.2 ml of extracting solution (80% methanol with 0.01 mol/L butylated hydroxytoluene (BHT) and 0.1% formic acid). The mixture was vortexed for 10 s and ground for 2 min, followed by ultrasonic extraction for 2 h and centrifuging at 12,000 rpm for 10 min. The precipitation was extracted again with ultrasonic and centrifuging. All the supernatants were vacuum freeze-dried and then diluted with 100 µl 80% methanol for UPLC-ESI-MS/MS analysis.

Ultraperformance liquid chromatography (UPLC) analyses were performed using a Waters Acquity Ultraperformance Liquid Chromatography system (Milford, USA), equipped with a binary pump system (Waters). The UPLC analyses were performed using an Acquity UPLC BEH C18 column (100 mm×2.1 mm i.d., 1.7 μm particle size) (Waters) with a binary mobile phase. Solvent A was methyl alcohol and B was water with 0.1% formic acid. The gradient elution was as follows: 0–1.5 min, 35–75% (v/v) A; 1.5–6 min, 75–95% (v/v) A; and 6–8 min, 95% (v/v) A; 8–8.1 min, 95-35% (v/v) A; 8.1–10 min, 35% (v/v) A. The flow rate was 0.8 ml/min and the sample volume injected was 5 μl. The UPLC system was coupled to the API4000 QTRAP mass spectrometer (Applied Biosystems, USA.) using a Z-spray electrospray ionization (ESI) source. The data were acquired in MRM mode with ion spray voltage: 4.5 kV, curtain gas (nitrogen): 35 psi, ion source gas 1: 30 psi, ion source gas 2: 45 psi, turbo gas temperature: 550°C. Samples were examined using multiple reactant monitoring with the (m/z) precursor/product ion information in **Supplemental Table S4**. Peak integration on the major isomer was performed using Analyst 1.6.2 software (Applied Biosystems, USA).

### Expression Network Construction

Hierarchical cluster analyses were separately performed for the PC genes [mean fragments per kilobase of transcript per million mapped reads (FPKM) ≥ 2] using the OmicShare tools (www.omicshare.com/tools). WGCNA (v1.47) was used to construct the unsigned co-expression networks based on the transcript expression matrix. A step-by-step network construction and module detection method were adopted using the “cutreeDynamic” and “mergeCloseModules” with the following parameters: the power was 13; the minModuleSize was 30; the cutHeight was 0.25. We investigated the relationships between the transcripts in the modules and the samples, and the important modules that were significantly associated with the content of flavonoid, formononetin, isoliquiritigenin, genistein, and catechin. To understand the biological functions of the modules, the genes in the modules were subjected to GO enrichment analysis. Finally, the co-expression network was visualized by Cytoscape (v3.5.0) software.

### Yeast One-Hybrid Assays

One-hybrid system in YM4271 yeast strain was used to test the binding ability of MYBs to the promoter of *DFR, LAR, IFSs*. Promoter fragments were inserted into pLacZi as reporters. *MYBs* were expressed in the yeast cells with pGADT7-AD. Total DNA was isolated from fresh young leaves of 8-year-old *S. suberectus* using the Plant DNA Kit (TIANGEN) according to the manufacturer’s instructions. The extraction of messenger RNA (mRNA) was performed with the Oligotex mRNA Mini Kit (Qiagen). Long-distance PCR (LD-PCR) was executed by the PCR cDNA Synthesis Kit (SMART). The promoters cloned from the DNA template and the full-length cDNA of two MYBs were cloned from the cDNA library. All the detail of these sequences were provided in the **Supplemental Data S1**. The primers were listed in **Supplemental Table S5**.

## Results

### Comparative Genome Analysis and Divergence Time Estimation

**Figure 2:**
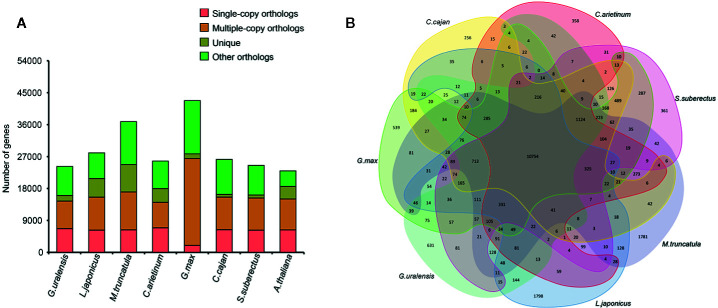
Comparative analyses of *Spatholobus suberectus* with other plants. **(A)** The gene number in four clusters of eight plant species. **(B)** Shared and unique gene families.

To investigate the evolution of *S. suberectus* genome, we compared it with the genome of seven other sequenced plant species (**Figure 2A**), namely, *G. max*, *L. japonicus*, *G. uralensis*, *Cicer arietinum*, *M. truncatula*, and *Cajanus cajan*, which are Leguminosae plants, and *Arabidopsis thaliana* as an outgroup. A total of 24,523 (77.5%) *S. suberectus* genes were clustered into four groups and included 853 unique genes, 6,253 single-copy orthologs. Overall, 108 single copy genes that were shared among eight angiosperm plants (**Supplemental Table S1**). A total of 361 gene families, consisting of 853 genes, were unique to *S. suberectus* (**Figure 2B**). The accuracy of these results were further validated the accuracy by the phylogenetic analysis. A total of 1,073 single-copy orthologs were obtained from eight species, indicating that the Leguminosae plants can be divided into galegoid (*M. truncatula*, five genes unique to *G. uralensis* and *C. arietinum*) and Millettioid (*G. max*, *C. cajan*, and *S. suberectus*) clades. *G. max* is much closer to *C. cajan* than *S. suberectus* within the Leguminosae family. We estimated the divergence times of *S. suberectus* from the other plants, and the results suggested that galegoid clade diverged from the Millettioid clade approximately 30.8 million years ago, and the divergence of *G. max*-*C. cajan* common ancestor and *S. suberectus* occurred approximately 18 million years ago (**Supplementary Figure 1**).

### Whole-Genome Duplication and Gene Family Expansion Analysis

**Figure 3:**
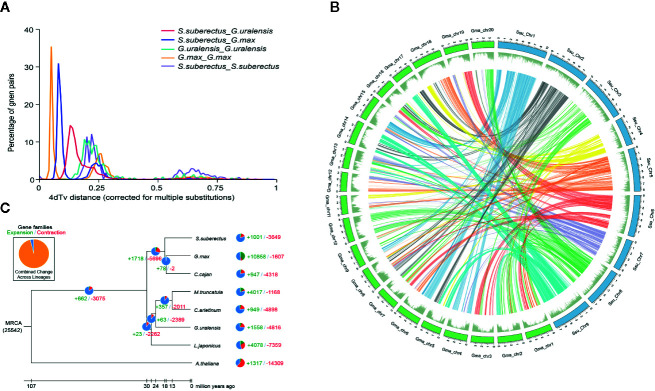
Whole-genome duplication and gene family expansion analysis. **(A)** Whole genome duplication (WGD) events detected in genome of *Spatholobus suberectus*, *Glycine max*, and *Glycyrrhiza uralensis*. 4dTv distribution of transversion substitutions at fourfold degenerate sites. **(B)** Circular diagram showing genetic collinearity between *S. suberectus* and *G. max.* Circles from inside to outside are as followed: a, the genome collinear blocks of between *S. suberectus* and *G. max*, which connected by curved lines and set as same color; b, gene density (green). All distributions are drawn in a window size of 300 kb, chromosomes_units = 500,000. **(C)** Gene family expansions and contractions in *S. suberectus* and seven other plants.

Whole-genome duplication (WGD) events are common in plant genomes and have a significant role in plant evolution and speciation. To investigate WGDs in the *S. suberectus* lineage, we identified the syntenic regions across the *S. suberectus*, *G. max*, and *G. uralensis* genomes. Each region consists of at least five collinear homologous genes. Two ancient whole-genome duplication (WGD) events occurred in these three species: γ event (all core eudicots share an ancient WGD, 4dtv [transversion substitutions at fourfold degenerate sites) =0.6] and a WGD incident shared by Leguminosae plant (4dtv=0.25), suggesting they occurred prior to their divergence. A third WGD event that occurred in *G. max* might have contribute to the divergence of *S. suberectus* and *G. max* (**Figure 3A**). The presence of 1,409 syntenic blocks between *S. suberectus* and *G. max* are present in the multiple copies in *G. max* strongly suggests that the *G. max* lineage underwent a WGD after speciation from *S. suberectus* (**Figure 3B**). The expansion and contractions of orthologous gene families were determined, and the result revealed 1,001 expanded gene families and 3,649 contracted gene families in the *S. suberectus* lineage (**Figure 3C**).

### Metabolic of Bioactive Flavonoid and Gene Families Involved in Flavonoid Biosynthesis

**Figure 4:**
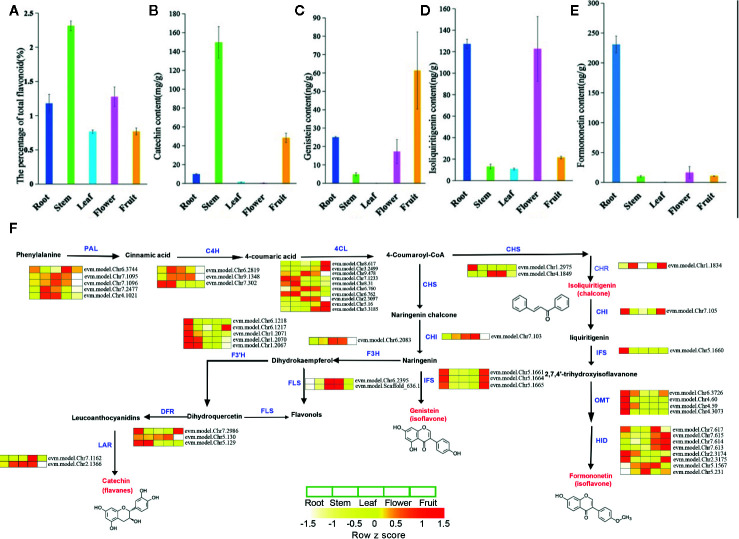
The metabolic profiles and detailed biosynthetic pathways of flavonoid of various tissues in *Spatholobus suberectus.* **(A)** The percentage of total flavonoid content. **(B)** The content of formononetin. **(C)** The content of isoliquiritigenin. **(D)** The content of genistein. **(E)** The content of catechin. **(F)** Detailed biosynthetic pathways of flavonoid in *S. suberectus.* The abbreviated name of enzyme in each catalytic step is showed in blue font. Gene expression levels [log_10_ (RPKM+1)] in five tissues are represented by color gradation. Gene expression with RPKM≤ 1 was set to 0 after log10 transformation. Genes with more than one homology are represented by equal colored horizontal stripe and are termed from top to bottom. The names of enzymes are listed as followed: PAL, C4H, 4CL, CHS, CHI, IFS, HID, F3H, F3’H, DFR, FLS, LAR, OMT, CHR. Each plant tissue for gene expression had three biological repetitions.

**Figure 5:**
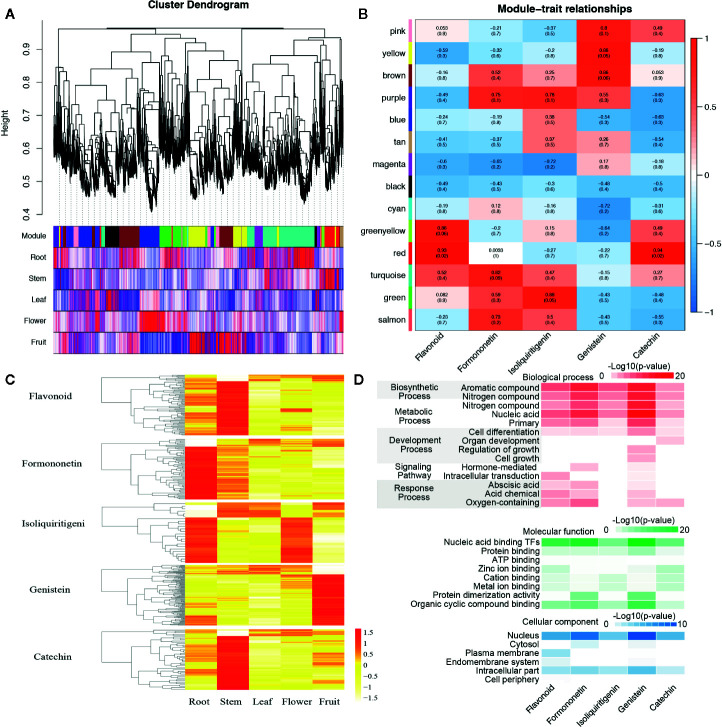
The co-expression networks of transcripts involved in the biosynthesis and metabolism of flavonoid, formononetin, isoliquiritigenin, genistein, and catechin. **(A)** Hierarchical cluster tree and color bands indicating the 14 modules identified by weighted gene co-expression network analysis (WGCNA). **(B)** The analysis of module–trait correlations. Each row represents a module and each column represents a specific chemical compound. Each cell at the row–column intersection is color-coded by correlation according to the color legend. **(C)** The expression abundance and cluster of flavonoid, formononetin, isoliquiritigenin, genistein, and catechin in roots, steams, leaves, flowers, and fruits. **(D)** Gene ontology (GO) enrichment analysis of genes belonging to the related cell of flavonoid, formononetin, isoliquiritigenin, genistein, and catechin.

We investigated the metabolic processes in various tissues of *S. suberectus* (**Figures 4A–E**). Flavonoids can be detected in all the tissues of *S. suberectus.* The quantitative metabolite profiles of total flavonoid compounds showed that the stem had the highest accumulation (up to 2.3%), root and flower tissues had moderate accumulation (~1%), and the other two tissues had minimum accumulations (**Figure 4A**). Formononetin and isoliquiritigenin are widely synthesized in the roots (**Figures 4E** and **5C**). Genistein can be detected in other tissues and is mainly synthesized in the fruit (**Figure 4C**). As a medicinal ingredient, the content of catechins in the stems of *S. suberectus* is more abundant than other flavonoids (Li et al., 2017) and may be the reason that the stem had the highest amount of flavonoids (**Figure 4B**).

In this study, through comparative analysis with homologous genes of *Arabidopsis*, soybean, and other reported plants, we have searched 14 gene families involved in flavonoid synthesis in the *S. suberectus* genome (**Supplemental Tables S2** and **S3**). The detailed biosynthetic pathways of the flavonoid in various tissues are shown in **Figure 4F**. Based on their expression levels in five tissues, all of them had at least one highly expressed member in all the tissues (**Figure 4F** and **Supplementary Table S2**). Notably, the number of *PAL*, *4CL*, and *CHS* homologs in *S. suberectus* was dramatically decreased relative to *G. max* (**Supplementary Table S2**).

The different expression trends of genes in different tissues may determine the difference in the content of flavonoids in these tissues. The high flavonoid content in *S. suberectus* tissues is likely due to the constant and high expression of flavonoid biosynthesis-related genes. *IFS* catalyzes the oxidation of naringenin to genistein and plays an important role in the biosynthesis of formononetin. Gene expression profiling and RNA-seq data mining showed that *IFS* (chr5.1661; chr5.1664; chr5.1665) copies have maintained their transcriptional activity in root and fruit tissues, and *IFS* (chr5.1660) is highly expressed in the root. These results indicated that organ-specific expression patterns are similar to those observed in different formononetin and genistein synthesis pathways in different tissues (**Figures 4C, E**).

### Differential Expression Networks of the Biosynthesis and Metabolism of Kinds of Flavonoid

Flavonoid content in percentage varies among the stems, leaves, and other tissues. For the exploration of genes related to the biosynthesis and metabolism of different flavonoids, a weighted gene co-expression network analysis (WGCNA) was performed with the RNA-Seq and the content data, 14 distinct modules were obtained and shown in the dendrogram (**Figure 5A**). The modules were labeled with different colors and correlated with the content of flavonoid, and formononetin, isoliquiritigenin, genistein, and catechin were of particular interest in our study. We found the “greenyellow” and “red,” “turquoise,” “green,” “yellow” and “brown,” “red” modules are significantly associated with the content of flavonoid, formononetin, isoliquiritigenin, genistein, and catechin respectively (**Figure 5B**). It is worth noting that genes in the “greenyellow” and “red” modules were highly expressed in the stem, the module “turquoise” comprised transcripts that were highly expressed in the root, while genes which related to the content of genistein were highly expressed in the fruits, and parts of genes were also highly expressed in the roots (such as the *IFS*). These results were merely coincidental with the genistein biosynthetic pathways of various tissues in *S. suberectus* in **Figure 4C**. And the gene oncology (GO) classification of genes related to the content of genistein primarily showed an involved in the aromatic compound (genistein contains an aromatic A ring) biosynthetic process (such as *PAL1*, Chr7.1096/Chr7.1095), the metabolic of nucleic acid, and a certain amount of genes were nuclear localization transcription factors (such as *WRKY*, Chr8.1000; *bHLH*, Chr6.3388; *MYB*, Chr8.323/Chr9.1209 et al.) (**Figure 5D**). All these results showed that *IFS* plays an important role in the biosynthesis of flavonoids.

### Gene Expansion Involved in Flavonoid Biosynthesis

**Figure 6:**
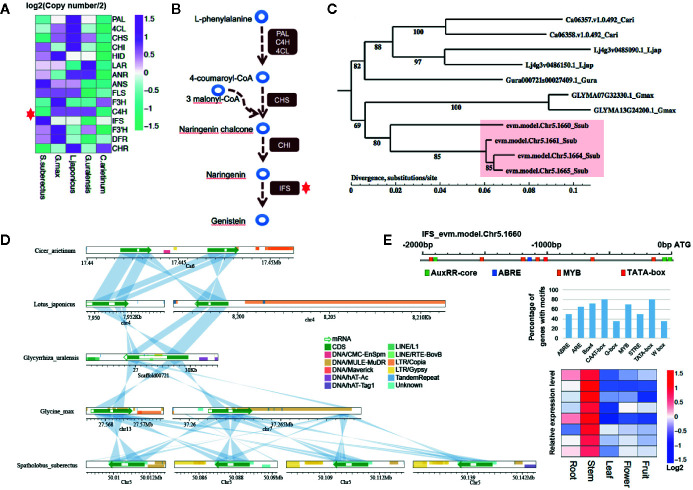
Gene family analysis showing expansion in isoflavonoid biosynthesis genes involved in the biosynthesis of genistein and formononetin compounds. **(A)** Two gene families involving in the biosynthesis of flavonoid were expanded in *Spatholobus suberectus*, including the anthocyanidin synthase (ANS) and isoflavone synthase (IFS) family. **(B)** IFS is the key enzyme for biosynthesis of isoflavones, which catalyzes 5,7,4’-trihydroxyflavanone (naringenin) to genistein. **(C)** Phylogeny of the *IFS* genes in *S. suberectus*, *Glycine max*, *Glycyrrhiza uralensis*, *Cicer arietinum*, and *Lotus japonicus* showing four copies of *S. suberectus* *IFS* genes. Numbers correspond to branching posterior probabilities. *IFS* genes are upregulated in the stem and root of *S. suberectus*. The heatmap of *IFS* genes expression is corresponding to the order of *IFS* genes in the tree. **(D)** The four genomic regions in chromosome five containing the *S. suberectus*. *IFS* genes show clear synteny with the *G. max* genome, while the four *IFS* genes occur in tandem. This suggests the involvement of both whole-genome duplication (WGD) and tandem duplication events in *IFS* family expansion. Lines linking the two bars indicate regions with >70% similarity and coverage length >=100. (**E**) The arrangement of cis-elements on the promoter of *IFS* (Chr5.1660), the percentage of genes with different cis-elements in their promoter regions and the expression profiles of *MYBs* related to the biosynthesis of flavonoid.

To further explain how *IFS* affects the content of flavonoid scientifically, the expansion of the gene families involved in flavonoid biosynthesis were analysis. Interestingly, *IFS* expanded compared with other Leguminosae species (red star, four copies in *S. suberectus*, two copies in *G. max*, *C. arietinum* and *L. japonicus*, and one copy in *G. uralensis*; **Figure 6A**). Studies on Leguminosae showed that *IFSs* are the key enzyme for the biosynthesis of genistein (**Figure 6B**). To investigate potential mechanisms of the expansion of *IFS* from a single copy in the Leguminosae ancestor to four copies in *S. suberectus*, we performed phylogenetic analysis in *IFS* in Leguminosae (**Figure 6C**). The four *S. suberectus* *IFSs* were in close proximity on the same chromosome and presented on two separate chromosomes in *G. max*. In addition, *IFS* had only one copy in *G. uralensis* (**Figure 6C**). This result suggests that *IFS* initially expanded in the *G. max* lineage through a large-scale genomic duplication event (such as a WGD). Nevertheless, the different copies of *IFS* in *S. suberectus*, *C. arietinum*, and *L. japonicus* underwent unequal tandem duplication events. Microsynteny analysis provided clear evidence that *IFS* genes in Leguminosae showed regional synteny to each other (**Figure 6D**). All the results demonstrated that *IFSs* in Leguminosae evolved by lineage-specific whole-genome and tandem duplications. We found that they are difference in the presence of retrotransposon in the position of the *IFS* homologue in Leguminosae (**Figure 6D**). Two *G. max* *IFS* genes all had overlap with DNA transposons (DNA/MULE-MuDR) in exon, however, *IFS* of *G. uralensis* was inserted by LINE/RTE-BovB in the intron position. One of *C. arietinum* *IFS* (Ca06358.v1.0.492) had a little overlap with LTR/Copia at the end of the second exon. *L. japonicus* and *S. suberectus* *IFS* genes had no internal insertion by repetitive element, but a large number of repetitive element distributions were observed before (the promoter regions) or after these genes (**Figure 6D**).

### Identification of the Upstream Regulatory Transcription Factors of Flavonoid Biosynthesis

Given that a certain number of genes correlated with the content of flavonoid are transcription factors, motif discovery with the promoter regions (from −2,000 to 0 bp to the ATG) of genes participating in the flavonoid biosynthesis (163 genes) were performed using MEME-ChIP. Except the TATA-box (ATATATAT, E-value=3.6–012) and CAAT-box (CAAT, E-value=2.7–009), which cis-acting sequence elements are generally found upstream of the ATG, the binding sites of MYB TF families (CAACA/CG/A) showed the highest occurrences frequency with the E-value of 5.5e−007. The promoter region of *IFS* (*Chr5.1660*) had only one ABRE site (cis-acting element involved in the abscisic acid responsiveness), three AuxRR-core sites (cis-acting regulatory element involved in auxin responsiveness), and six MYB TFs binding sites (**Figure 6E**). Five of MYB TF binding sites were obtained by the insertion of DNA/MULE-MuDR. On the other hand, all the MYB TFs in the “yellow” and “brown” modules were specific highly expressed in the stem (**Figure 6E**), that were also coincidence with the stem has the high percentage of total flavonoid in the stem (**Figure 4A**). In general, we preliminarily speculated the activated retrotransponson positive regulate the accumulation of flavonoid in *S. suberectus* by introducing the cis-elements of TFs specifically expressed in the stem (such as MYBs).

**Figure 7:**
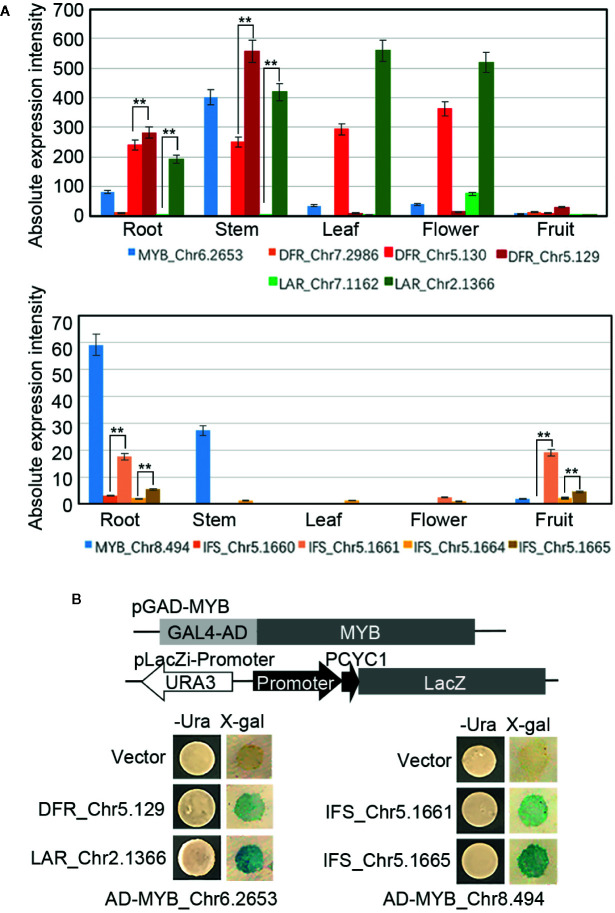
Regulate networks of the MYBs. **(A)** Expression profiles of *MYBs, DFRs, LARs*, and *IFSs* in the roots, stems, leaves, flowers, and fruits. **(B)** Promoter fragments of *DFR, LAR*, and *IFSs* were connected to pLacZi and transformed into YM4271 strain harboring GAL4-AD-MYBs. The β-galactosidase activity was validated using X-gal staining. All bars represent means± s.d, and three biological replicates in the experiment. Significant differences (Student’s t-test) at P < 0.01 (**).

To further identify the crucial MYB TFs and the candidate downstream genes of them, two MYB TFs (Chr6.2653 and Chr8.494) (**Figure 7A**), whose expression (based on the RNA-seq analysis results) were significantly correlated with the content of flavonoids or catechin respectively, were cloned and recombined in the pGADT7-AD as the effectors. Meanwhile, one *DFR* (Chr5.129) and one *LAR* (Chr2.1366) whose expression were highly correlated with the content of flavonoids, two *IFS* (Chr5.1661 and Chr5.1665) whose expression were highly correlated with the content of catechin (all of these four genes with higher absolute expression intensity than other homologous genes (**Figure 7A**) and with the binding motifs of the MYB TFs in their promoter regions (**Supplemental Data S1**)) were cloned and recombined into the pLacZi. A one-hybrid yeast assay in YM4271 strain proved that AD-*MYB_*Chr6.2653 interacted with pLacZi-*DFR_*Chr5.129*/LAR_*Chr2.1366, and AD-*MYB_* Chr8.494 interacted with pLacZi-*IFS_*Chr5.1661/Chr5.1665 (**Figure 7B**), which identified these two MYB TFs respectively recognize the promoter regions of their candidate target genes *in vitro* and *MYB_*Chr6.2653/Chr8.494 played roles in the biosynthesis of flavonoids/catechin by regulating the expression of key genes in the synthetic pathway.

## Discussion

### The Revolution of *Spatholobus suberectus* and the Genome Expansion

Our previous study provided a high-quality genome sequence for *S. suberectus* (Qin et al., 2019). It is worth noting that the genome size of *S. suberectus* (748 Mb) is smaller than the *Glycine max* (1.1 G) but bigger than the *G. uralensis* (379 M) (Mochida et al., 2017). Basing on the genomic data published on National Center for Biotechnology Information (NCBI), we discovered three WGD events in *G. max* (**Figure 3A**, yellow line), WGD analysis also identified *S. suberectus* underwent two WGD events (**Figure 3A**, purple line) and the recent WGD event happened before the latest WGD event in *G. max*. Meanwhile, 47.82% of the *S. suberectus* genome was occupied by the repetitive elements and 17.32% of the genome the long terminal repeat (LTR), both of them were lower than that of *G. max* genome (53.9% were repetitive elements, 34.1% were LTR) (Qin et al., 2019; Xie et al., 2019). *G. uralensis* also underwent two WGD events (**Figure 3A**, green line), and the proportion of transposable elements and unclassified repeats was only 36.48% in *G. uralensis* (Mochida et al., 2017). Taken together, these evidences showed the transposable element (TE) insertion resulted in the larger genome size of *S. suberectus* than *G. uralensis*, on the other hand, both of the WGD and the expansion of the repetitive sequence (especially the LTR) were the great contributors to the genome expansion of *G. max.*

### Correspondence Analysis Between the Flavonoids Content and the Key Gene Expression Profiles

To further identified the crucial genes for the biosynthesis of flavonoids, the RNA-seq results analyses were combined with the content of flavonoids. Formononetin was specific accumulated at the root, that was just correlated with the root specific expression profiles of *CHI_*Chr7.105*, IFS_*Chr5.1660*, OMT_*Chr6.3726/4.60/4.59/4.3073, and *HID_*Chr2.3174/2.3175. The content of genistein was highly correlated with the root and fruit specific expressed *IFSs* (Chr5.1661/5.1004/5.1665*)*. But, one worth noting thing was the content of isoliquiritigenin showed no clear correlation with the expression of its upstream synthesis regulatory genes such as the *CHS* (high expressed in the leaf and flower) and *CHR* (high expressed in the stem and fruit). That might have related to isoliquiritigenin being an intermediate, which content was not only determined by the expression of upstream biosynthesis regulatory genes, but also the metabolism of intermediate. About the accumulation of catechin, *DFR_* Chr5.129 showed the highest absolute expression intensity at the stem, that was just coincidence with the highest content of catechin in the stem. *DFR*_Ch7.2986 and *LAR*_Chr7.1162 showed on low expression profile at all the tissues and *DF*R_Chr5.130 and *LAR*_Chr2.1366 highly expressed in all the tissues excepted the flower. It can be speculated that *DFR*_Chr5.12*9* is a dominant gene involved in the biosynthesis of catechin than other homologues genes, and *DF*R_Chr5.130 and *LAR*_Chr2.1366 might play important roles in the vegetative development.

### The Expansion of Isoflavone Synthase Accelerated the Biosynthesis of Flavonoids in *Spatholobus suberectus*

*S. suberectus* has been widely used as the traditional medicines plant source because it contained various flavonoids, which was higher than other Leguminosae plants. In particular, stems and roots of *S. suberectus* were choosed into medicine to treat anemia in the minority areas of China, it may be relevant to flavonoids as the main bioactive components with the similar gene expression pattern in these two tissues. Gene tandem duplication was one of the most intriguing features of the *S. suberectus* genome. Gene tandem duplication contributed to the development and environment adaption of different plants, such as the Leguminous plants nucleotide-binding site-leucine-rich repeat (NBS-LRR) genes were duplicated to improve the tolerance to kinds of diseases in the soybean (Shao et al., 2014) and most TCP, cytochrome P450, and NB-ARC domain gene families were derived from tandem duplication events in the genome of *Antirrhinum majus* L. to affect the development of flowers and abiotic stress response (Li et al., 2019). With the high-quality genome sequence for *S. suberectus*, a series of flavonoid-related genes were identified in *S. suberectus* genome compared to model plants. *IFS* was the key enzyme for the biosynthesis of genistein and 2,7,4’-trihydroxyisoflavanone (the substrate of formononetin) (**Figure 4F**), and these *IFSs* expression patterns were just highly coincidence with the consent of genistein in roots and fruits (**Figure 4C**), and formononetin in roots (**Figure 4E**). We identified four tandemly duplicated *IFS* genes (**Figure 6C**) with high sequence similarity and further synteny block assay indicated these four *IFSs* are tandemly located in Chr 5 (**Figure 6D**) and with high sequence similarity. Collectively, *IFSs*, the biosynthesis pathway of genistein and formononetin and generated by the tandem duplication, might strengthen the active control of the accumulation of kinds of flavonoids in the stem and root of *S. suberectus*.

### The Crucial Roles of MYB TFs in the Flavonoids Biosynthesis Regulation

TFs play a major role in regulating the genes expression in plant secondary metabolism, and the overexpression of TFs regulates the expression of multiple genes in biosynthetic pathways. Notably, MYB TFs, which is proved to be widely involved in plant phenylpropanoid metabolic pathways and plays an important regulatory role in plant flavonoid biosynthesis in various species such as peach and buckwheat (Matsui et al., 2018; Cao et al., 2019), were the most abundant TF family in *S. suberectus*. *PbMYB12b* could activate other genes involved in flavonoid metabolism and promote flavonoid synthesis (Zhai et al., 2019). In our study, over 70% genes, which participate in the flavonoid biosynthesis, had the MYB binding sites in their promoter regions. This results verified the intermediate regulatory roles of MYB TFs in the flavonoid biosynthesis pathway. Further, the cis-acting elements which recognized by stem-express MYB TFs were introduced by the activated retrotransponson: *MYB*_Chr6.2653 could recognize promoter regions of *DFR*_Chr5.129 and *LAR*_Chr2.1366, *MYB*_Chr8.494 could bind to promoter regions of *IFS*_Chr5.1661 and Chr5.1665 in the yeast. The expression of these two MYBs were correlated with the content of catechin or genistein respectively, and had higher absolute expression intensity than other homologous genes in the stem or root. All these results demonstrated the crucial roles of MYB TFs in the flavonoids biosynthesis regulation at the molecular level.

In summary, our study provided abundant valuable information on the genomic resources of *S. suberectus*, one of most important Chinese medicine plant. The genome and transcriptome data we provided here should be valuable to both fundamental biological research and breeding research. Therefore, on the basis of this study, the germplasm resources of *S. suberectus* will be further optimized for the alleviation of resources shortage.

## Data Availability Statement

Transcriptome data of root and stem tissues are available at https://figshare.com/articles/dataset/Transcriptome_data_of_different_Spatholobus_suberectus_tissues-1/12762473 (https://doi.org/10.6084/m9.figshare.12762473.v1).

The transcriptome data of fruit, flower and leaf are available at https://figshare.com/articles/dataset/Transcriptome_data_of_different_Spatholobus_suberectus_tissues-1/12780515 (https://figshare.com/articles/dataset/Transcriptome_data_of_different_Spatholobus_suberectus_tissues-1/12780515).

## Author Contributions

JM, CL, and ZZ designed the project. SQ and CL analyzed data and wrote the paper. SQ, KW, and YL performed experiments. ZC, XZ, CY, and LL contributed samples, materials, or data. ML, LG and WX helped with the data analysis and examined the results.

## Funding

This study was supported by the Guangxi science and technology research project (2020GXNSFBA159006, AB16450012, AA18242040), the National Public Welfare Special Project of China “Quality Guarantee system of Chinese herbal medicines” (201507002), the China Agriculture Research System (CARS-21), “Guangxi Bagui Scholars” and Research Innovation Team Project (GuiYaoChuang2019005).

## Conflict of Interest

The authors declare that the research was conducted in the absence of any commercial or financial relationships that could be construed as a potential conflict of interest.

## Supplementary Material

Supplemental Figure S1 Estimation of the divergence time of *S. suberectus* and other 7 plant species.

Supplemental Data S1 Sequencing results of the ORFs or the promoter regions used in the one-hybrid yeast assay.

## References

[B1] Bowerman P. A. Ramirez M. V. Price M. B. Helm R. F. Winkel B. S. J. (2012). Analysis of T-DNA alleles of flavonoid biosynthesis genes in Arabidopsis ecotype Columbia. BMC Res. Notes 5, 485–493. 10.1186/1756-0500-5-485 PMC3526476 22947320

[B2] Cao Y. Xie L. Ma Y. Ren C. Xing M. Fu Z. (2019). PpMYB15 and PpMYBF1 Transcription Factors Are Involved in Regulating Flavonol Biosynthesis in Peach Fruit. J. Agric. Food Chem. 67, 644–652. 10.1021/acs.jafc.8b04810 30525549

[B3] Chen S. R. Wang A. Q. Lin L. G. Qiu H. C. Wang Y. T. Wang Y. (2016). In vitro study on Anti-Hepatitis C Virus Activity of Spatholobus Suberectus Dunn. Molecules 21, 1767–1383. 10.3390/molecules21101367 PMC6274077 27754461

[B4] Devic M. Guilleminot J. Debeaujon I. Bechtold N. Bensaude E. Koornneef M. (1999). The BANYULS gene encodes a DFR-like protein and is a marker of early seed coat development. Plant J. 19, 387–398. 10.1046/j.1365-313X.1999.00529.x 10504561

[B5] Dhaubhadel S. McGarvey B. D. Williams R. Gijzen M. (2003). Isoflavonoid biosynthesis and accumulation in developing soybean seeds. Plant Mol. Biol. 53, 733–743. 10.1023/B:PLAN.0000023666.30358.ae 15082922

[B6] Edgar R. C. (2004). MUSCLE: A multiple sequence alignment method with reduced time and space complexity. BMC Bioinf. 5, 113-131. 10.1186/1471-2105-5-113 PMC517706 15318951

[B7] Gupta S. Nawaz K. Parween S. Roy R. Sahu K. Kumar Pole A. (2017). Draft genome sequence of Cicer reticulatum L., the wild progenitor of chickpea provides a resource for agronomic trait improvement. DNA Res. 24, 1–10. 10.1093/dnares/dsw042 27567261 PMC5381347

[B8] Hashim M. F. Hakamatsuka T. Ebizuka Y. Sankawa U. (1990). Reaction mechamism of oxidative rearrangement of flavanone in isoflavone biosynthesis. FEBS Lett. 271, 219–222. 10.1016/0014-5793(90)80410-K 2226805

[B9] Jung W. Yu O. Lau S. M. C. O’Keefe D. P. Odell J. Fader G. (2000). Identification and expression of isoflavone synthase, the key enzyme for biosynthesis of isoflavones in legumes. Nat. Biotechnol. 18, 208–212. 10.1038/72671 10657130

[B10] Li L. Stoeckert C. J. Roos D. S. (2003). OrthoMCL: Identification of ortholog groups for eukaryotic genomes. Genome Res. 13, 2178–2189. 10.1101/gr.1224503 12952885 PMC403725

[B11] Li J. Li C. Gou J. Wang X. Fan R. Zhang Y. (2016). An alternative pathway for formononetin biosynthesis in Pueraria Lobata. Front. Plant Sci. 7 , 861–873. 10.3389/fpls.2016.00861 PMC4905983 27379141

[B12] Li M. Liu J. Luo D. Hua P. Wu Z. Han Z. (2017). Correlation of Flavonoids Content of Caulis Spatholobi with Soil Nutrients. Trad. Chin. Drug Res. Clin. Pharmacol. 2, 238–243. 10.19378/j.issn.1003-9783.2017.02.020

[B13] Li M. Zhang D. Zhang H. Gao Q. Ma B. Chen C. (2019). Genome structure and evolution of Antirrhnum majus L. Nat. Plants 5, 174–183. 10.1038/s41477-018-0349-9 30692677 PMC6784882

[B14] Matsui K. Oshima Y. Mitsuda N. Sakamoto S. Nishiba Y. Walker A. R. (2018). Buckwheat R2R3 MYB transcription factor FeMYBF1 regulates flavonol biosynthesis. Plant Sci. 274, 466–475. 10.1016/j.plantsci.2018.06.025 30080636

[B15] Mochida K. Sakurai T. Seki H. Yoshida T. Takahagi K. Sawai S. (2017). Draft genome assembly and annotation of Glycyrrhiza uralensis, a medicinal legume. Plant J. 89, 181–194. 10.1111/tpj.13385 27775193

[B16] Pelletier M. K. Shirley B. W. (1996). Analysis of flavanone 3-hydroxylase in arabidopsis seedlings: Coordinate regulation with chalcone synthase and chalcone isomerase. Plant Physiol. 111, 339–345. 10.1104/pp.111.1.339 8685272 PMC157841

[B17] Pelletier K. Murrell J. R. Shirley B. W. (1997). Characterization of Flavonol Synthase and Leucoanthocyanidin Dioxygenase Genes in Arabidopsis. Plant Physiol. 113, 1437–1445. 10.1104/pp.113.4.1437 9112784 PMC158268

[B18] Peng F. Meng C. W. Zhou Q. M. Chen J. P. Xiong L. (2016). Cytotoxic Evaluation against Breast Cancer Cells of Isoliquiritigenin Analogues from Spatholobus suberectus and Their Synthetic Derivatives. J. Nat. Prod. 79, 248–251. 10.1021/acs.jnatprod.5b00774 26690274

[B19] Qin S. Wu L. Wei K. Liang Y. Song Z. Zhou X. (2019). A draft genome for Spatholobus suberectus. Sci. Data 6, 1–10. 10.1038/s41597-019-0110-x PMC6609623 31273216

[B20] Saito K. Yonekura-Sakakibara K. Nakabayashi R. Higashi Y. Yamazaki M. Tohge T. (2013). The flavonoid biosynthetic pathway in Arabidopsis: Structural and genetic diversity. Plant Physiol. Biochem. 72, 21–34. 10.1016/j.plaphy.2013.02.001 23473981

[B21] Saslowsky D. Winkel-Shirley B. (2001), Localization of flavonoid enzymes in Arabidopsis roots. Plant J. 27, 37–48. 10.1046/j.1365-313x.2001.01073 11489181

[B22] Sato S. Nakamura Y. Kaneko T. Asamizu E. Kato T. Nakao M. (2008). Genome structure of the legume, Lotus japonicus. DNA Res. 15, 227–239. 10.1093/dnares/dsn008 18511435 PMC2575887

[B23] Schmutz J. Cannon S. B. Schlueter J. Ma J. Mitros T. Nelson W. (2010). Genome sequence of the palaeopolyploid soybean. Nature 463, 178–183. 10.1038/nature08670 20075913

[B24] Schoenbohm C. Martens S. Eder C. Forkmann G. Weisshaar B. (2000). Identification of the arabidopsis thaliana flavonoid 3’-hydroxylase gene and functional expression of the encoded P450 enzyme. Biol. Chem. 381, 749–753. 10.1515/BC.2000.095 11030432

[B25] Shao Z. Q. Zhang Y. M. Hang Y. Y. Xue J. Y. Zhou G. C. Wu P. (2014). Long-term evolution of nucleotide-binding site-leucine-rich repeat genes: Understanding gained from and beyond the legume family. Plant Physiol. 166, 217–234. 10.1104/pp.114.243626 25052854 PMC4149708

[B26] Shimada N. Sasaki R. Sato S. Kaneko T. Tabata S. Aoki T. (2005). A comprehensive analysis of six dihydroflavonol 4-reductases encoded by a gene cluster of the Lotus japonicus genome. J. Exp. Bot. 56, 2573–2585. 10.1093/jxb/eri251 16087700

[B27] Shimamura M. Akashi T. Sakurai N. Suzuki H. Saito K. Shibata D. (2007). 2-Hydroxyisoflavanone dehydratase is a critical determinant of isoflavone productivity in hairy root cultures of Lotus japonicus. Plant Cell Physiol. 48, 1652–1657. 10.1093/pcp/pcm125 17921150

[B28] Shirley B. W. Hanley S. Goodman H. M. (1992). Effects of ionizing radiation on a plant genome: Analysis of two arabidopsis transparent testa mutations. Plant Cell 4, 333–347. 10.1105/tpc.4.3.333 1354004 PMC160133

[B29] Tanner G. J. Francki K. T. Abrahams S. Watson J. M. Larkin P. J. Ashton A. R. (2003). Proanthocyanidin biosynthesis in plants. Purification of legume leucoanthocyanidin reductase and molecular cloning of its cDNA. J. Biol. Chem. 278, 31647–31656. 10.1074/jbc.M302783200 12788945

[B30] Wang D. X. Liu P. Chen R. Y. Chen M. L. Chen G. Y. (2008). Effect of monomers extracted from Spatholobus suberectus Dunn on proliferation of hematopoietic progenitor cells in marrow-depressed mice. J. Clin. Rehabil. Tissue Eng. Res. 12, 4163–4166. 10.3321/j.issn:1673-8225.2008.21.006

[B31] Wang H. Liu Y. Zenc Z. He W. (2011). Study on HPLC chromatographic fingerprint of anti-tumor active site SSCE of Caulis spatholobi. Zhongguo Zhongyao Zazhi 36, 2525–2529. 10.4268/cjcmm20111816 22256759

[B32] Xie M. Chung C. Y. L. Li M. W. Wong F. L. Wang X. Liu A. (2019). A reference-grade wild soybean genome. Nat. Commun. 10, 1216. 10.1038/s41467-019-09142-9 PMC6418295 30872580

[B33] Young N. D. Debellé F. Oldroyd G. E. D. Geurts R. Cannon S. B. Udvardi M. K. (2011). The Medicago genome provides insight into the evolution of rhizobial symbioses. Nature 480, 520–524. 10.1038/nature10625 22089132 PMC3272368

[B34] Zhai R. Zhao Y. Wu M. Yang J. Li X. Liu H. (2019). The MYB transcription factor PbMYB12b positively regulates flavonol biosynthesis in pear fruit. BMC Plant Biol. 19, 85–95. 10.1186/s12870-019-1687-0 PMC6385385 30791875

[B35] Zhou Z. Y. Huan L. Y. Zhao W. R. Tang N. Jin Y. Tang J. Y. (2017). Spatholobi Caulis extracts promote angiogenesis in HUVECs in vitro and in zebrafish embryos in vivo via up-regulation of VEGFRs. J. Ethnopharmacol. 200, 74–83. 10.1016/j.jep.2016.10.075 27989880

