# Supplementary figures and images for: Comparative Genomics of Spatholobus suberectus and Insight Into Flavonoid Biosynthesis
Source: Front Plant Sci. 2020 Sep 4;11:528108. doi: 10.3389/fpls.2020.528108 (PMC7500164; doi:10.3389/fpls.2020.528108)

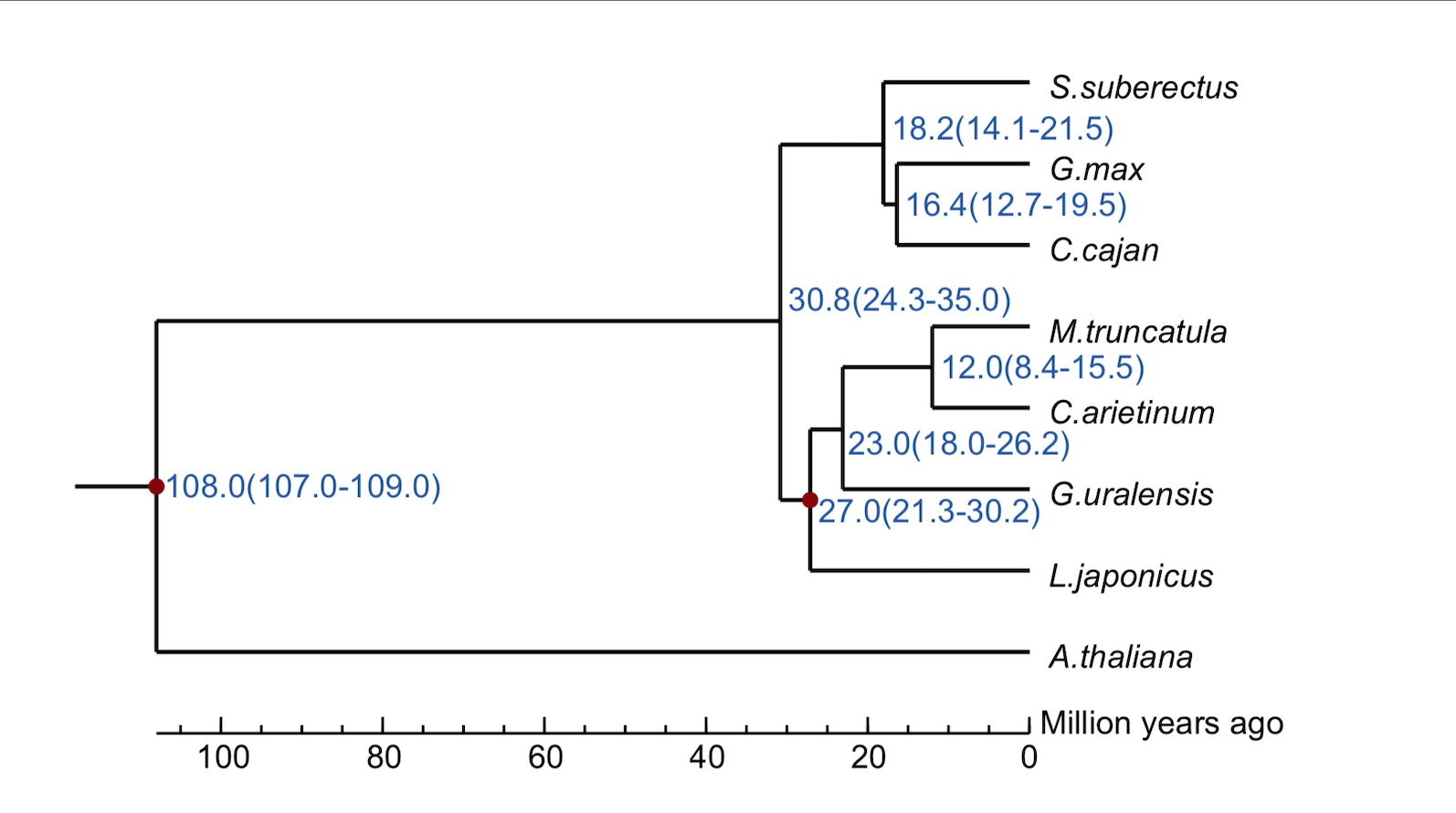

Supplement: Supplemental Figure S1 — Estimation of the divergence time of S. suberectus and other 7 plant species. [file Image_1.jpeg]
