# Supplementary material for: Comparative Genomics of Spatholobus suberectus and Insight Into Flavonoid Biosynthesis
Source: Front Plant Sci. 2020 Sep 4;11:528108. doi: 10.3389/fpls.2020.528108 (PMC7500164; doi:10.3389/fpls.2020.528108)
Supplement: Supplemental Data S1 — Sequencing results of the ORFs or the promoter regions used in the one-hybrid yeast assay. [file Table_1.docx]

**Supplemental Table 1: The gene number in four cluster of 8 plant species**

| **Species** | **Single-copy orthologs** | **Mutiple-copy orthologs** | **Unique** | **Other orthologs** |
| --- | --- | --- | --- | --- |
| *G.uralensis* | 6695 | 7745 | 1573 | 8202 |
| *L.japonicus* | 6260 | 9294 | 5216 | 7297 |
| *M. truncatula* | 6338 | 10669 | 7748 | 12110 |
| *C.arietinum* | 6922 | 7171 | 3847 | 7805 |
| *G.max* | 1963 | 24500 | 1328 | 14992 |
| *C.cajan* | 6395 | 9164 | 767 | 9892 |
| *A.thaliana* | 6327 | 8711 | 3533 | 4378 |
| ***S.suberectus*** | 6253 | 9054 | 853 | 8363 |

**Supplemental Table 2: The ID of flavonoid biosynthesis genes in *S.suberectus***

| **Gene** | **ID** |
| --- | --- |
| PAL | evm.model.Chr6.3744 |
|  | evm.model.Chr7.1095 |
|  | evm.model.Chr7.1096 |
|  | evm.model.Chr7.2477 |
|  | evm.model.Chr4.1021 |
| C4H | evm.model.Chr6.2819 |
|  | evm.model.Chr9.1348 |
|  | evm.model.Chr7.302 |
| 4CL | evm.model.Chr8.617 |
|  | evm.model.Chr3.2499 |
|  | evm.model.Chr9.478 |
|  | evm.model.Chr7.1233 |
|  | evm.model.Chr8.31 |
|  | evm.model.Chr6.760 |
|  | evm.model.Chr6.762 |
|  | evm.model.Chr2.3097 |
|  | evm.model.Chr5.16 |
|  | evm.model.Chr3.3185 |
| CHS | evm.model.Chr1.2975 |
|  | evm.model.Chr4.1849 |
| CHR | evm.model.Chr1.1834 |
| CHI | evm.model.Chr7.105 |
|  | evm.model.Chr7.103 |
| OMT | evm.model.Chr6.3726 |
|  | evm.model.Chr4.60 |
|  | evm.model.Chr4.59 |
|  | evm.model.Chr4.3073 |
| HID | evm.model.Chr7.617 |
|  | evm.model.Chr7.615 |
|  | evm.model.Chr7.614 |
|  | evm.model.Chr7.613 |
|  | evm.model.Chr2.3174 |
|  | evm.model.Chr2.3175 |
|  | evm.model.Chr5.1567 |
|  | evm.model.Chr5.231 |
| F3H | evm.model.Chr6.2083 |
| FLS | evm.model.Chr6.2395 |
|  | evm.model.Scaffold_636.1 |
| F3'H | evm.model.Chr6.1218 |
|  | evm.model.Chr6.1217 |
|  | evm.model.Chr1.2071 |
|  | evm.model.Chr1.2070 |
|  | evm.model.Chr1.2067 |
| DFR | evm.model.Chr7.2986 |
|  | evm.model.Chr5.130 |
|  | evm.model.Chr5.129 |
| LAR | evm.model.Chr7.1162 |
|  | evm.model.Chr2.1366 |
| IFS | evm.model.Chr5.1660 |
|  | evm.model.Chr5.1661 |
|  | evm.model.Chr5.1664 |
|  | evm.model.Chr5.1665 |

**Supplemental Table 3: The number of flavonoid biosynthesis related genes in each plant species**

| **Gene** | ***S.suberectus*** | ***G.max*** | ***L.japonicus*** | ***G.uralensis*** | ***C.arietinum*** |
| --- | --- | --- | --- | --- | --- |
| PAL | 5 | 8 | 22 | 8 | 4 |
| 4CL | 12 | 16 | 22 | 12 | 8 |
| CHS | 5 | 17 | 24 | 16 | 6 |
| CHI | 5 | 4 | 6 | 4 | 3 |
| HID | 10 | 12 | 9 | 9 | 6 |
| LAR | 2 | 2 | 1 | 4 | 1 |
| OMT | 17 | 19 | 13 | 13 | 12 |
| FLS | 4 | 4 | 4 | 1 | 2 |
| F3H | 2 | 4 | 2 | 2 | 3 |
| C4H | 3 | 5 | 5 | 5 | 3 |
| IFS | 4 | 2 | 2 | 1 | 2 |
| F3'H | 5 | 6 | 4 | 5 | 3 |
| DFR | 4 | 6 | 3 | 2 | 3 |
| CHR | 1 | 3 | 10 | 3 | 7 |

**Supplemental Table 4:** **Mass spectrometry parameters of the target components**

| **Component** | **RT(min)** | **Precursor ion (m/z)** | **Product ion (m/z)** | **DP/V** | **CE/V** | **CXP/V** |
| --- | --- | --- | --- | --- | --- | --- |
| Catechin | 3.39 | 289.0 | 245.0 | -79 | -21 | -9 |
|  |  |  | 109.1 | -80 | -31 | -3 |
|  |  |  | 203.0 | -86 | -27 | -9 |
| Genistein | 4.83 | 269.0 | 133.0 | -109 | -45 | -10 |
|  |  |  | 107.0 | -115 | -42 | -3 |
|  |  |  | 158.8 | -188 | -42 | -5 |
| Isoliquiritigenin | 5.02 | 255.0 | 119.2 | -75 | -31 | -8 |
|  |  |  | 134.9 | -77 | -23 | -8 |
|  |  |  | 91.2 | -80 | -41 | -13 |
| Formononetin | 5.43 | 267.0 | 251.9 | -104 | -30 | -13 |
|  |  |  | 223.1 | -102 | -45 | -9 |
|  |  |  | 132.0 | -100 | -44 | -4 |

**Supplemental Table 5: Primers used in this study**

| **Gene** | **ID** | **Primer F** | **Primer R** |
| --- | --- | --- | --- |
| MYB | evm.model.Chr6.2653 | ATGAAGTGGGAAACTATAACC | CTAGTGTTGTGAAGACGGTTG |
| DFR | evm.model.Chr5.129 | CTACTGCATCTCCAACGGGG | AGCTTCATGTCTCCATTGCCT |
| LAR | evm.model.Chr2.1366 | TGGTCAATCTTGGAGGCAGG | CGATGGGCAGAGCTACTTGA |
| MYB | evm.model.Chr8.494 | ATGATTCAACAGGAAGTGCG | TTAGCCGGTAAAGAATACGT |
| IFS | evm.model.Chr5.1661 | ACTTCCACAGTCCGCACAAA | CACTATGCCACGGGACTTGT |
| IFS | evm.model.Chr5.1665 | GTTTGCTATGAATCTCTGCCCA | GCAACTTCGCTTCAGACCAC |
