# Supplementary material for: Comparative Genomics of Spatholobus suberectus and Insight Into Flavonoid Biosynthesis
Source: Front Plant Sci. 2020 Sep 4;11:528108. doi: 10.3389/fpls.2020.528108 (PMC7500164; doi:10.3389/fpls.2020.528108)
Supplement: Supplementary file 3 [file Table_2.docx]

>DFR_Chr5.129 promoter

CTGGTTGCTAGGGTGCTGTAGTTTTTTTATGAGGGTGGACTTTATTTGGGGCTTTCTCTTCTCTTATCTTATTTTATTGGCTTTGTTAAAGGATGGTAATTAGGGATGGCAAAATGGGTCTTTTCATTTCTCTCAACCATTAGATTAATTCAATGACGCACATTTTTGCTAGAGAGTTGGACAGAAATTTAGGCCAGAGAGGATCTGAATGTTGTGTGGAGTGCATGTAATAACCTTAAGTTATTTACTTATTTTCATCTAGAAAGCTACATTCTTGCCGCTGATGATTATTTTCTTAAAGGAAACTCCCGTAGACTCAATGAAAACATCAGTGTTGAAGCTATGCAACCTTCAAGCGCCATCCAGCGAGGAGCTAGCCTAATGCTTGGAATCTTCTTCAACAACTTTGGTATAGTATTTGTGCACTTTTACATTGGCACTACCTGTGCAACAGTTTGTGCATGCATGATATAATCTCAGAATTACCTTATTTGATGACGATCGCATCGGTGTTACTGAGAAATTGACTCTAATTGGTCTTTAGTTTTGGGACTTAGGAGCAACATCAACATTACTTTTTGTCCAGAGTCTCGGTATTTTAACTCACGTTTCCTTTCTTAATTATCAACTTTTATCAATCTCAGTACTACCACATATCACATGTTCTCATGTATGAATATTAATTTCACACATGAAAGAACCATTATTAGTCGTTTAATAAATAACCATTATTATTAAGACGCAATTAAAAGCAATTTCCGACATAATGATTAGTGAGAAGTGACAAAAAAATAATCAAGAGGCAATGGAGACATTAAGCTGGTACCCGGGGATCTGTCGACCTCGAGGCATGTGCTCTGTATGTATATAAAACTCTTGTTTTCTTCTTTTCTCTAAATATTCTTTCCTTATACATTAGGTCCTTTGTAGCATAAATTACTATACTTCTATAGACACGCAACACAAATACACACACTAAATTAATAATGACCGGGATCCGGAGCTTGGCTGTGCCCGTCTCACTGGTGAAAGAAAAACACCCTGGGCGCCCATTACGCAAACCGCCTCCTCCCCGCCGCGTTG

>LAR_Chr2.1366 promoter

GGCAGCCTCGGATCGGTCATTATTAATTTAGTGTGTGTATTTGTGTTTGCGTGTCTATAGAAGTATAGTAATTTATGCTACAAAGGACCTAATGTATAAGGAAAGAATATTTAGAGAAAAGAAGAAAACAAGAGTTTTATATACATACAGAGCACATGCCTCGAGGTCGACAGATCCCCGGGTACCCGATGGGCAGAGCTACTTGAAGGATTTCATAAGTTTTTAATTTTTCTTTTGAAGATTAACTTAGGACCAATTGTAATTTAGATATAAATAGACATTCTTAGGGATTTTATTTTTATCTTTTGAACTCTATTGGAAGCATAAAGAATTGGGGGATTTTCTTTCTTTTCTTTTTGGTATTCTCTCATAACTATGTATGAGTAGAACCTTATTTTTCTAGGATTCGACGTAGCCCTACGATTGTCTTAATGAATTAGTTTTTGCTTACCTTTGTATGATCTTGTTCCTCTTTTGATTCTTTTCTATTGATTTCTCTATGCAAAGCAAATTGGCCAACGTTTTTCATAACCTTAGGGATTTGGGGAAGTATAGAAAGGTGAACCCAATTATCGAACCCATAGGAAAGGAACTTAGGTTTAAACCACGAAAGTAGGCTAAAACCCTCGAGGATTAATTGAATTAACAAAGAACTTATTGGGTCTAATTAAACACAGCCATCACGATAGTAAGTGGTAAATTTGGTTAAATACCCCTTCATATCTTGAAAGAGAACAAAGGGTACCTTAGGTTAATTCACATTCAATAAGAACAAGTGAAACACAAAGGTGGTGGAATTAAGGCATTAAGCGTCATAGTGTGAAACCCTAATCTTAGAAAGCTTCTCATCTTGAATTTCTCCTAATTTTTATTGTTGTTTGATTTAATCTTTAATTTCTCTCAATTGCTTTACCCTGTATTTTCCCAATTAAATTTTTTTTGTTTAA

>IFS_Chr5.1661 promoter

TTTTCGGGAATACGAATCAAAAAATTTCAAGGAAACCGAAATCAAAAAAAAGAATAAAAAAAAAATGATGAATTGAAAAGCTTGAATTCGAGCTCGGTACCACTTCCACAGTCCGCACAAATTATCAAGTTGTCAAATTCAAATGTATAAGGATAGGTCACTAATGTATATAAAAATAAATAAAAAAATGCTAGGTCACCAATGTTTGAGTTGAGAATATTGTTACTTTTTTCAAGTGTGAGATGAAAATGCACGTTCCTCCTGTCATGATGGACATTTAAATTTCTTCTTGACAAAATGCCACGAACATCGTTAGCTAAAGTTCGTACTATTAAAATACAAGTCCCGTGGCATAGTGGGTACCCGGGGATCTGTCGACCTCGAGGCATGTGCTCTGTATGTATATAAAACTCTTGTTTTCTTCTTTTCTCTAAATATTCTTTCCTTATACATTAGGTCCTTTGTAGCATAAATTACTATACTTCTATAGACACGCAAACACAAATACACACACTAAATTAATAATGACCGGATCCGGAGCTTGGCTGTTGCCCGTCTCACTGGTGAAAAGAAAAACCACCCTGGCGCCCAATACGCAAACCGCCTCTCCCCGCGCGTTGGCCGATTCATTAATGCAGCTGGCACGACAGGTTTCCCGACTTAATCGCCTTGCAGCACATCCCCCTTTCGCCAGCTGGCGTAATAGCGAAGAGGCCCGCACCGATCGCCCTTCCCAACAGTTGCGCAGCCTGAATGGCGAATGGCGCTTTGCCTGGTTTCCGGCACCAGAAGCGGTGCCGGAAAGCTGGCTGGAGTGCGATCTTCCTGAGGCCGATACTGTCGTCGTCCCCTCAAACTGGCAGATGCACGGTTACGATGCGCCCATCTACACCAACGTGACCTATCCCATTACGGTCAATCCGCCGTTTGTTCCCACGGAGAATCCGACGGGTTGTTACTCGCTCACATTTAATGTTGATGAAAGCT

>IFS_Chr5.1665 promoter

TGTTCCGGGAAATACGTCAAAAATTTCAAGGAAACCGAAATCAAAAAAAAGAATAAAAAAAAAATGATGAATTGAAAAGCTTGAATTCGAGCTCGGTACCGGTTGCTATGAGTCTCTGCCCATTTTGTTCCTTTGTTTATCCTTTGGATGATTTTATATTCTTCCGTAATATTTTTTATTTATTAAATAAAAATCTTAATAGGTATTTTTAAGAAATTATTTCATGAAAAAAATTAAAAATATGAAATAGAAATTTTATTAATTACCGATAAATTCATCGCTATCTGAAAGTAATTTATAAGGTTCGTGCATTATAACAATTTCATCTTCTTAAAATTATAAAAAGACATGTAAATATTATCTTGAATTTTGGACTACTTACATAATTAATAAAAAATTTATTGATTTAAGTTATATTTACTATAAAACGAATTAGCTTTAATAATTTTCTCATAAAATTTATAATCTTAATTGGCAATCCCATACCAGCTAAGATTTATAAACTTTTATAAATATTGTAATTTTATTTAATATTATCTATTTCCACATATAATATTATGTTGTATATCCAAAAAAAAAAAATGCTAACGACCAAGGTGTGAGTTGATAATATTGTTACTTTATTTTCAAGTAGGAGATGATAATGCACGATCATCCTGTCATGATGGATAGTTAAATTTCTTCTTGACTTTATGACGAGAACATCGATAGCGATAGTTCTTACTATTATCATACAATCCCTGTGGCTAGTGGCATCGTGGGTCTGAATTCGCTGTTGCCTAGTACACGTGGGATCCTAACGCCCTAATACGCAGACCGACGTACTCCTACGCAGCGTAGTCGAATACATATATCGACTTGAACTGAGCCACGCACTAGGGGTCTTACCGGTACGTATTAATCTCCGCACCCA

>MYB_ Chr6.2653

ATGAAGTGGGAAACTATAACCCTCTCTCCTGCACCTTCTACTCCCAACTCCAACACCAGTTGGTTGGTGGAAGATAATAGGAGCACAAAATGGACCCCTGAAGAGAACAAGCTGTTTGAGAATGCTCTTGCAGTGCATGATAAGGACACCCCAGATCGGTGGCACAAAGTGGCTGAAATGATTCCAGGAAAGACTGTAGGGGATGTGATCAGGCAGTACGAGGAGTTGGAAGCGGATGTTAGCAATATAGAAGCTGGGTTGATCCCAGATCCTGGCTATAGTACTGCCACCTCACCACCCTTCACCTTAGATTGGGTGAACACTCCTGGTTATGATGGGTTTAAAGGAAGTGGTAAGAGATCTTCCTCAGTGAGACCTCATGAGCAGGAAAGGAAGAAAGGAGTGCCTTGGACTGAAGAGGAACATAAATTGTTTCTACTGGGTCTGAAGAAGTATGGGAAAGGTGATTGGAGAAATATTTCTCGCAATTTTGTGATTACTAGAACCCCAACACAGGTTGCCAGCCATGCTCAGAAGTACTTCATAAGGCAACTTTCAGGAGGCAAAGACAAGAGGAGGGCTAGCATACATGACATAACAACAGTGAATCTCACTGAAACCACCACAACTTCTTCGTCAGAAGACACCAATAGATCCACTTCACCACATGTGCTCTCACAGCAGCAGCCAAATTCTACTGCCACTACACCCACAACTCATTTTCAGTGGAGCAATCAGTCAAACACAGGAGTAGCTATGACTCTCAATCCTGCTAATGAAAGAGTCTTCATGTCTCCTTATGGTGCTAACTCCTTTGGGGTTAAAATGCAGGGACAGAATCTGCACAAGACTGCTCTTCATGAGTCTTCTTACTTGGGACCTCAGGCACAGAACATGGTTTTCCAAATGCAACCGTCTTCACAACACTAG

>MYB_ Chr8.494

ATGATTCAACAGGAAGTGCGCAAAGGTCCGTGGACAGAACAGGAAGACTTCAAATTGGTGTCCTTTGTTGGCTTGTTTGGAGACCGTAGATGGGACTTTATAGCTAAGGTATCAGGTTTGAATAGAACAGGTAAGAGTTGCAGGTTACGGTGGGTTAATTACCTCCATCCTGGCCTCAAACGAGGGAAGATGACAACCCAGGAAGAGCGCCTTGTGTTGGAGCTTCACTCAAAATGGGGAAATAGGTGGTCAAGAATTGCGCGCAAATTACCAGGGCGCACTGACAATGAGATTAAGAATTATTGGAGAACTCTGATGAGGAAAAAGGCTCAGGACAAGAAGCGAGGAGAAGCTGCATCACCAGCTCCTTCTAGTGTTCATTCCTCAATTTCCTCAAACAACCATGCAGTGGATCCACATGCTTCCAAAAAGGCTGGAGAAGAGAGCTTTTATGACACAGGAGGTCCTGGTATGATAGGCTCAACCCAAGATCAGGGTCAGAAAGGTGAACAAGGGTTCTCTATGGATGATATATGGAAAGATATTGACATATCAGAAGGGAACACTCTGCAGCCAGTGTATGATGGGCACAGTGAAGATGGCTGCAACTTCTCTTGCCCACAAGTGCCTTCTCCATCGTGGGAGTATTCTTCTGAACCTCTATGGGTGATGGATGAGGAAAGTTTGTTTTGCCCCATGAGTGAACCATATTTTTCCTGCTATGCACAAGGCAACGTATTCTTTACCGGCTAA
